# Summer heat stress and strain during outdoor running in Aotearoa New Zealand

**DOI:** 10.1186/2046-7648-4-S1-A24

**Published:** 2015-09-14

**Authors:** Toby Mündel, Melissa Black, Nicole E Moyen, Blake Perry

**Affiliations:** 1 School of Sport and Exercise, Massey University, New Zealand; 2 Department of Health, Human Performance and Recreation, University of Arkansas, USA

## Introduction

A large body of work has identified the response to and consequence of exercise in a hot climate. However, typically this has been laboratory-based, which often misrepresents such a 'climate'. Those field studies conducted have often been based in the Northern Hemisphere during summer periods. Solar stress during a Southern Hemisphere summer, particularly New Zealand, is ~10 % greater for a given latitude in the Northern Hemisphere (or the combined effects of an equator-ward shift of 4° in latitude and 1 km increase in altitude) due to i) greater irradiances due to a lesser Sun-Earth separation owing to the elliptical orbit of the Earth about the Sun, ii) lower ozone levels due to the lesser Sun-Earth separation, and iii) lower tropospheric pollution [1].

The purpose of this study was to measure heat exposure and strain from running outdoors in the New Zealand summer, in order to determine what factor(s) might predict performance and risk for heat illness.

## Methods

Using an observational field study design, 10 male runners (age: 45 ± 12 y, peak speed: 19 ± 2 km.h^-1^) took part in a weekly 7 km race (start time 18:15 h) over six weeks (45 observations). Pertinent climatic measures were recorded including T_db_, T_wb _and T_bg_, and thus WBGT. Along with performance time, starting and end gastro-intestinal temperature (T_gi_), time-averaged arm temperature (T_arm_), and starting urine specific gravity were measured and a questionnaire asking about perception (thermal comfort, thermal sensation and exertion) and heat illness symptoms was administered following the race. Data were analysed using linear regression.

## Results

Of the six weeks observed, two weeks measured a WBGT above 27 °C; for both these weeks T_db _remained below 26 °C and relative humidity below 60 % (T_wb _≤ 20 °C) whilst T_bg _measured 53 and 57 °C, respectively. Urine specific gravity mostly (78%) described participants as not being dehydrated when beginning the race (i.e. <1.020). An end-T_gi _> 40 °C was observed on 10 occasions, however, few participants registered symptoms of heat illness and mean thermal sensation registered above "warm" only on three of the ten occasions and mean thermal comfort never registered above "uncomfortable." Linear regression identified perceived exertion and thermal comfort at the end of the race as strongest predictors of performance (model *R^2 ^*= 0.50, *p *= 0.02), T_db _and T_wb _as strongest predictors of end-T_core _(model *R^2 ^*= 0.38, *p *= 0.09), and performance time and starting T_gi _as strongest predictors of heat illness symptoms (model *R^2 ^*= 0.30, *p *= 0.37).

## Discussion

Based on the WBGT during the race, the most recent ACSM guidelines [2] would classify this climate as high risk for unfit, unacclimatized individuals; this is despite relatively low T_db _and T_wb _values, and highlights the increased role that solar radiation (T_bg_) plays to summer heat exposure in New Zealand. Nevertheless, regular runners achieve high end-T_core _(> 40 °C) without significant symptoms of heat illness. How hard runners push themselves (perceptually) and how thermally comfortable they are best predict performance time, whilst wet- and dry-bulb temperatures best predict the risk for heat illness.
